# A Preliminary Study on the Relationship between Arthropod Diversity and Vegetation Diversity in Four Contrasting Ecosystems in Hanthana Mountain Range of Sri Lanka, during the Post-Monsoon Dry Season

**DOI:** 10.1155/2023/7608236

**Published:** 2023-11-16

**Authors:** W. A. Manasee Weerathunga, A. M. Gihan Athapaththu, L. D. Amarasinghe

**Affiliations:** Department of Zoology and Environmental Management, Faculty of Science, University of Kelaniya, Dalugama 11600, Kelaniya, Sri Lanka

## Abstract

This study assesses the relationship between arthropod and vegetation diversity in four ecosystems with different types of vegetation, during a post-monsoonal season. We determined the arthropod diversity in vegetation surrounding an aquatic environment (AQ), a broad-leaved wet, evergreen forest ecosystem (BL), a *Pinus caribaea* monoculture plantation (PN), and a *Pinus* plantation artificially enriched with indigenous broad-leaved tree species (PNEN) located in the Hanthana mountain range, Sri Lanka. Arthropods randomly sampled from three randomly selected sites (5 m × 5 m) of each ecosystem were identified up to the highest possible taxa using standard identification keys. Woody and herbal vegetation was identified via a plant census. Arthropod and vegetation diversities were computed separately for each site using the Shannon–Wiener Index (H). Arthropods of 68 species and 43 families were found. AQ had the greatest arthropod diversity (*H* = 2.642), dominated by *Olios* spp., followed by BL (*H* = 2.444), dominated by a tettigonid species, *Oxytate* spp. and *Psechrus* spp. PN was third (*H* = 1.411), dominated by *Dicaldispa* spp. PNEN had the lowest (*H* = 1.3500), dominated by an ant species. Contrastingly, PNEN had the highest plant diversity (*H* = 2.614) and PN, the lowest (*H* = 0.879). In AQ, BL, and PN, the arthropod diversity was linearly dependent on plant diversity (*R*^2^ = 0.423, *p* ≤ 0.001), whereas it was not so when PNEN was also included (*R*^2^ = 0.008, *p* ≤ 0.001). This shows that higher plant diversity contributes to greater arthropod diversity in ecosystems where human intervention is minimal. But this pattern was not visible in PNEN, which is an artificially created ecosystem.

## 1. Introduction

Like all other animals, arthropods have a strong interrelationship with their surrounding vegetation, with herbivorous arthropods and their associated trophic levels playing a major role. Several studies have identified plant diversity as an important determinant of arthropod diversity [1, 2]. Several models and experimental studies [3–5] bear evidence for the increase of arthropod diversity with increasing plant diversity. There are several hypotheses to explain this phenomenon [6, 7]. Resource specialization hypothesis (RSH) and more individual hypothesis (MIH) are two of those hypotheses which have gained prominence. According to RSH, approximately, 90% of herbivorous arthropods show host-specific specialization [8]. Therefore, as the plant species richness increases, the number of associated herbivore species should also increase accordingly [9]. In contrast, MIH states that if above-ground net primary productivity (ANPP) increases as plant species richness increases, then more herbivore individuals will be supported owing to the increased availability of resources [10]. Therefore, an increased number of herbivore species by either of these hypotheses shall support more predator species [11–13].

However, as Ebeling et al. [3] point out, most of these classical hypotheses are seldom focused on entire communities or ecosystems, but focal species. Therefore, the influence of vegetation diversity may be different for the diversity of arthropods in different species groups and trophic levels, depending on the ecological roles played by different arthropods in the community. Stronger positive correlations between arthropod and vegetation diversity have been shown for primary than secondary consumers [5]. On the other hand, Haddad et al. [2] showed that species richness of both herbivore and predator arthropods were strongly positively correlated to plant species richness. Notably, Haddad et al. [2] found that arthropod species richness shifts from a predator-dominated trophic structure to an herbivore-dominated structure with decreased plant species richness. Interestingly, the diversity of herbivore arthropods has been shown to be more strongly related to diversities of predators and parasites than to plant diversity [14]. Also, Prather et al. [15] show that although arthropod diversity can be constrained by plant richness and abiotic conditions such as droughts cause deviations from that pattern.

Architectural or structural diversity of plants, probably correlating with their functional and species diversity, could determine arthropod diversity [16, 17]. Meanwhile, changing plant diversity can play a major role in interactions between herbivorous arthropods and their predators and parasites [2, 3].

Hanthana mountain range, the study area of the present research, is located within the humid tropical climatic zone in the Central Highlands of Sri Lanka. It traverses an altitudinal range from *ca.* 500 to 800 m above mean sea level, displaying substantial spatial variation in plant and ecosystem diversity. The different ecosystems present within the Hanthana mountain range include tropical broad-leaved wet evergreen forests, interspersed with grasslands and monoculture *Pinus carribaea* plantations. Historical evidence suggests that broad-leaved wet evergreen forests were the dominant vegetation type in this area. Improper land management has led to the dominance of perennial grasslands in this area, posing a serious threat to the ecosystem stability of the entire area.

As a means of restoring the soil and vegetation and increasing biodiversity, indigenous tree species were planted by partial removal of *Pinus* [18]. This has given rise to a mixed forest plantation. Numerous works elsewhere in the world, where vegetation diversity had been increased or decreased artificially, report subsequent increases or decreases in arthropod diversity [19, 20]. However, there has been no previous work to determine whether the same has happened in *Pinus* plantations “enriched” with indigenous tree species (termed “enriched” *Pinus*, PNEN) in the Hantana mountain range. In fact, except for studies targeting a particular group of arthropods [21], a general survey on arthropod diversity has not been carried out in this area in the recent past. The objective of this study is to provide baseline data on the arthropod diversity of the Hanthana mountain range and its interaction with plant diversity.

Therefore, in the present work, which was intended to be a preliminary short-term investigation, our objectives were to find answers to the following questions: (a) Do the different ecosystems that are present within the Hanthana mountain range show significant variation in their arthropod diversity? (b) If so, is there evidence to support the generally established positive relationship between arthropod diversity and vegetation diversity? (c) Has the artificial increase of vegetation diversity in the monoculture *Pinus* plantations via enrichment planting of indigenous tree species resulted in an increase in arthropod diversity after two decades?

The Hanthana mountain range has been an area of severe human intrusion during the past four decades. However, there are no previous studies on the human impacts on the diversity of arthropods of this region. We believe that the present work will provide valuable information, which can be part of plans and programmes to conserve biodiversity and ecosystem stability in the Hanthana mountain range.

## 2. Methodology

### 2.1. Study Area

Hanthana mountain range (7°15′ N, and 80°37′ E) is located in the mid-country wet zone of Sri Lanka, and is divided into two major regions, namely, upper Hanthana mountain area (>600 m) and lower Hanthana mountain area (<600 m). It has been identified that the lower Hanthana area is subjected to heavy human encroachment and upper Hanthana area is comparatively pristine [21]. This study was carried out during the months of August-September, 2016 in a dry season following the South-Western monsoons.

### 2.2. Experimental Design

**Figure 1:**
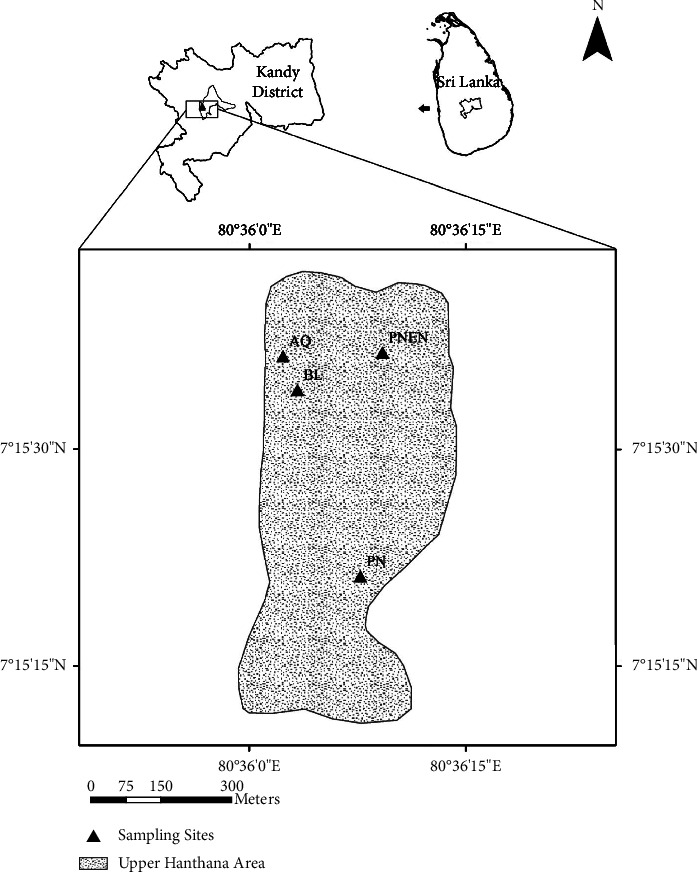
Map of study area showing sampling sites aquatic vegetation (AQ), broad leaved vegeation (BL), *Pinus* monoculture (PN), and enriched *Pinus* vegetation (PNEN).

Four contrasting ecosystems located close to each other in Hanthana mountain range, were selected as different treatments of the study. The four ecosystems were, namely, vegetation surrounding an aquatic environment (AQ), a broad-leaved, wet evergreen ecosystem (BL), *Pinus caribaea* monoculture vegetation (PN), and a *Pinus* plantation artificially enriched with broad-leaved tree species (PNEN). In each of these ecosystems, three replicate sites (5 m × 5 m) were chosen randomly and temporarily demarcated. AQ and BL treatments were located in the lower Hanthana area while PN and PNEN treatments were located in upper Hanthana area (Figure 1).

AQ was a flatland bordered by a manmade water body on one side. BL ecosystem was an area with a slight inclination and a thick growth of broad-leaved wet evergreen tree species forming a dense canopy, in contrast to PN where *Pinus caribaea* was the dominant tree species with a *Panicum maximum* dominated grassland as undergrowth. PNEN was a land that had been a *Pinus caribaea* monoculture previously but had been enriched with artificially recruited broad-leaved species about 20–25 years ago.

### 2.3. Data Collection on Arthropods and Plants

**Figure 2:**
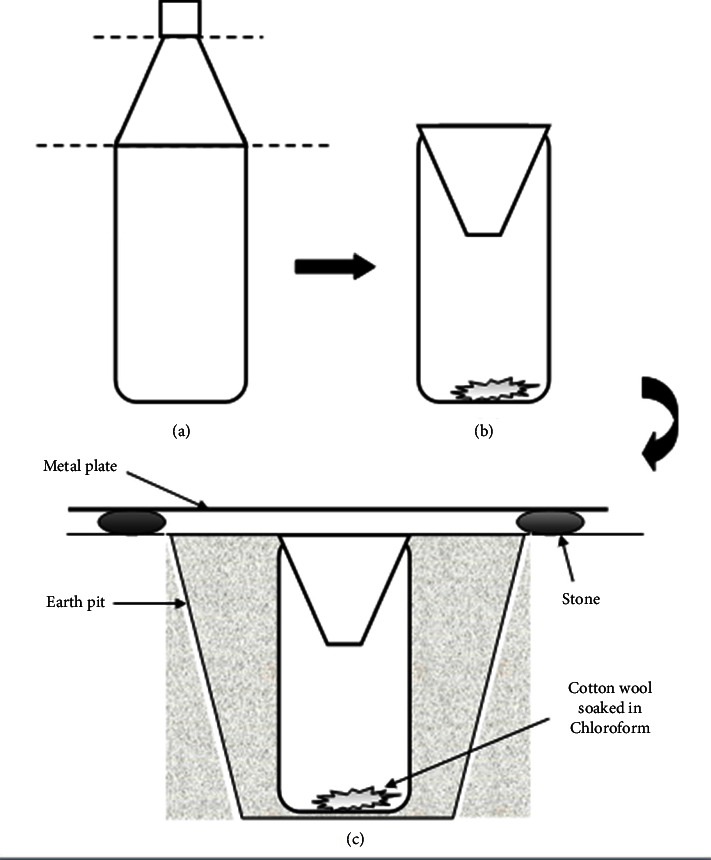
Schematic diagram of the way of preparing a pit-fall trap from a plastic water bottle (a) and (b) and as established on the ground (c).

Arthropods in each 5 m × 5 m replicate site were sampled using four different sampling methods (i.e. pit-fall traps, sticky traps, sweep net, and beating tray) in a way that arthropods at all heights from ground level are covered. Around 40–50 sweeps were done by the sweep net in each replicate site, for sampling arthropods at moderate heights above ground. A circular cloth having a diameter of 120 cm was used as the beating tray, which was held under unreachable foliage in the sampling plot, for sampling arthropods at unreachable heights above ground. Self-designed pit-fall traps and sticky traps were set for a period of one week, for sampling arthropods at ground level. Pit-fall traps were prepared by cutting a plastic water bottle of diameter in half. A piece of cotton wool soaked in chloroform (0.5 mL) was placed at the bottom of each pit-fall trap and they were covered with a metal sheet (Figure 2). Square plastic sheets (15 cm × 15 cm) spread with Vaseline were used as sticky traps, for sampling arthropods at mid-level above ground. Three pit-fall traps and three sticky traps were used for each replicate site. The traps were left for a period of one week and the arthropods collected were preserved in 70% alcohol and were identified to the highest possible taxa using standard identification keys, based on their morphological characteristics. Simultaneously, a plant census was also done for each replicate site to identify the plant species and their abundance. Every plant that was present in the sampling plot was individually identified using their morphological characteristics, with the help of standard pictorial guides and herbarium specimens.

### 2.4. Statistical Analysis

The diversity of arthropods and plants were calculated separately for each replicate site, using the Shannon–Wiener Index (H). Each replicate site consisted of a plot of 5 m × 5 m area. Species richness and species evenness was also calculated for each of those sites. The significance of variation between the four ecosystems, based on arthropod diversity was determined by one-way analysis of variation (ANOVA) for the diversity indices obtained for each replicate site of each ecosystem. Mean separation was done by using the least significant difference. The dependence of arthropod diversity on plant diversity was determined by conducting a simple linear regression analysis. The variation among the four ecosystems based on arthropod diversity and vegetation diversity was determined separately, using principle component analysis. All statistical analyses were carried out using Minitab 14.0 and Primer 5 software packages.

## 3. Results

**Figure 3:**
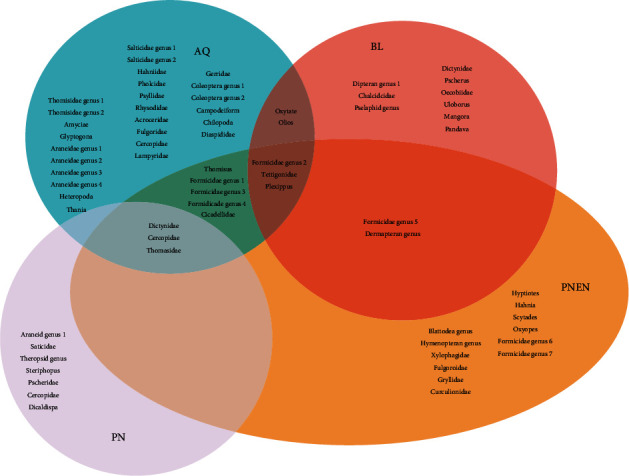
Venn diagram depicting arthropod families and genera found in each ecosystem.

Arthropod individuals belonging to 68 species and 43 families were collected from the 12 sampling sites (5 m × 5 m) across the four ecosystems. Arthropod genera and families found in each ecosystem is depicted in the Venn diagram given in Figure 3. In the same sampling area plant species belonging to 84 species and 42 families were enumerated.

**Figure 4:**
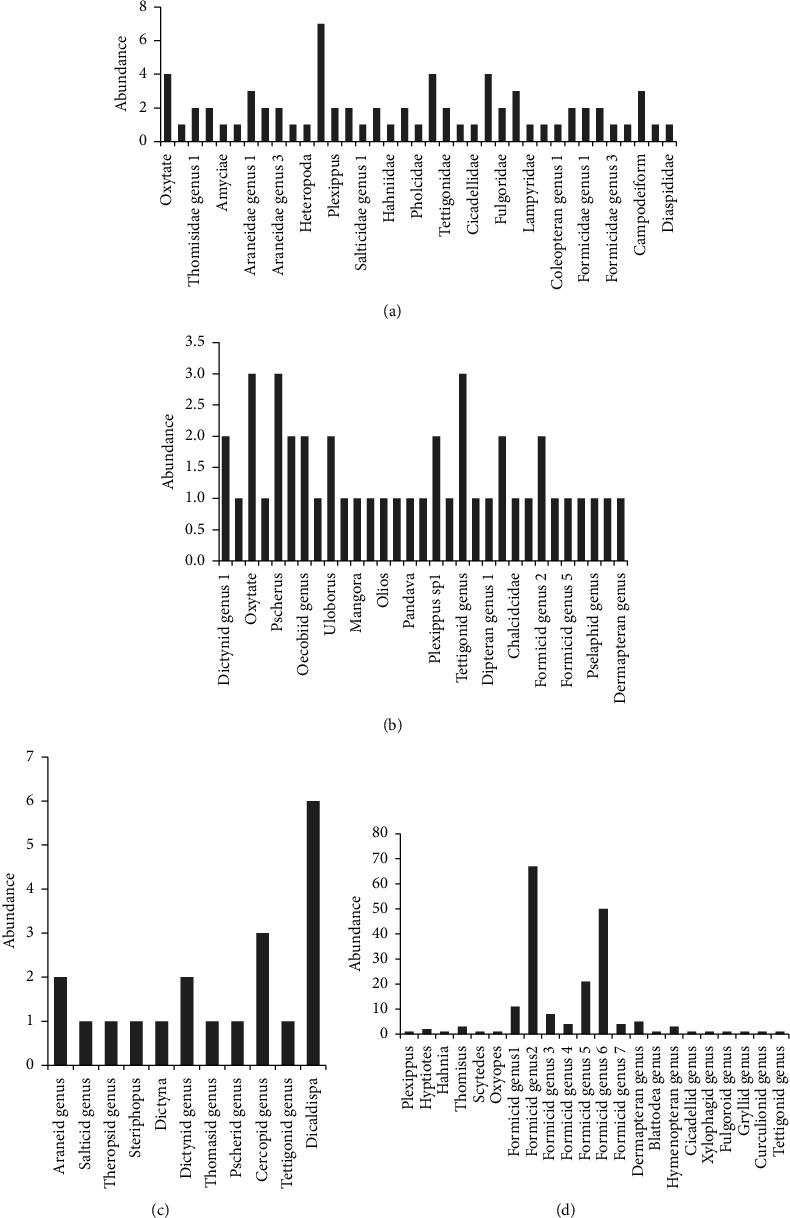
Arthropod diversity of (a). Aquatic-based vegetation (AQ), (b). Broad-leaved vegetation (BL), (c). Enriched *Pinus* vegetation (PNEN) (d). *Pinus monoculture*.

Based on the Shannon–Wiener Index (SWI), the vegetation surrounding the aquatic environment (AQ) had the highest arthropod diversity (SWI = 2.642) as well as the highest plant diversity. The most abundant arthropod found in this ecosystem was identified as *Olios* spp. (Araneae; Family Sparassidae) (Figure 4(a)). The second highest arthropod diversity (SWI = 2.444) was found in the broad-leaved, wet, evergreen ecosystem (BL). It was dominated by three arthropods, namely, a tettigonid species, *Oxytate* spp. (Araneae: Family Thomisidae, a crab spider genus), and *Psechrus* spp. (Family Psechridaea jungle cribellate spider genus) (Figure 4(b)). *Pinus caribaea* monoculture vegetation (PN) had the third highest arthropod diversity (SWI = 1.411) and it was dominated by *Dicaldispa* spp. (Coleoptera; Chrysomelidae) (Figure 4(d)). The lowest arthropod diversity (SWI = 1.3500) was found in the *Pinus* plantation artificially enriched with broad-leaved species (PNEN), which was dominated by an ant species (Hymenoptera; Formicidae) (Figure 4(c)). In contrast, when considering plant diversity, PNEN had the highest diversity (SWI = 2.614) and PN the lowest (SWI = 0.879). AQ (SWI = 1.810) and BL (SWI = 1.871) had intermediate values.

According to the ANOVA for the Shannon–Wiener diversity indices of each ecosystem, arthropod diversity was significantly higher (*p* < 0.1) in AQ and BL (Table 1) than in PN and PNEN. In contrast, the Shannon–Wiener Index for plant diversity was significantly (*p* < 0.01) the highest in the PNEN and was the lowest in PN while AQ and BL had intermediate values. Arthropod species richness showed significant (*p* < 0.001) variation among ecosystems with PN having a significantly lower value than the others, which did not differ significantly. In contrast, the ecosystems did not differ significantly in terms of plant species richness. The evenness of species distribution of both arthropods and plants showed significant variation among ecosystems (Table 1).

**Figure 5:**
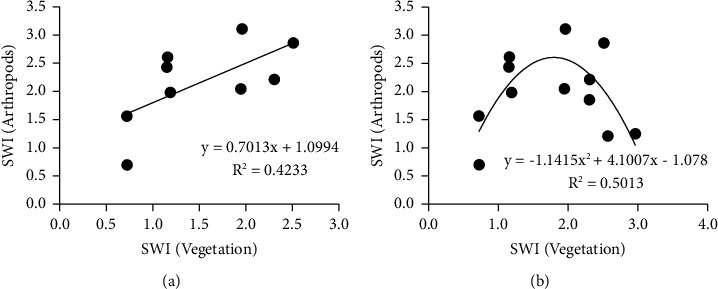
(a). Dependence of arthropod diversity on vegetation diversity for aquatic-based (AQ), broad-leaved (BL) vegetations, and *Pinus* monoculture (PN). (b). Dependence of arthropod diversity on vegetation diversity for all four ecosystems including enriched *Pinus* vegetation (PNEN).

When the diversity index data from ecosystems AQ, BL, and PN were included in a regression analysis, arthropod diversity displayed a significant (*p* < 0.05) positive linear dependence on plant diversity (Figure 5(a)). However, when the diversity data from PNEN were also included in the regression, a curvilinear dependence was observed (Figure 5(b)), where arthropod diversity decreased when vegetation diversity increased beyond a maximum.

**Figure 6:**
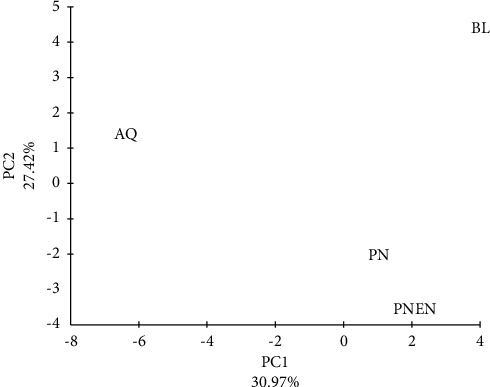
Variability of four ecosystems: aquatic-based vegetation (AQ), broad leaved vegetation (BL), *Pinus* monoculture (PN) and enriched *Pinus* vegetation (PNEN) depending on the principal component analysis carried out on the diversity of arthropod families recorded.

**Figure 7:**
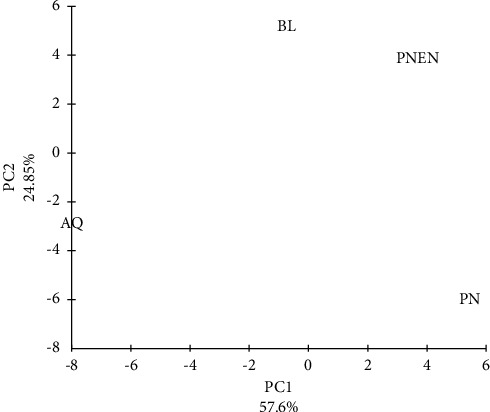
Variability among four ecosystems: aquatic-based vegetation (AQ), broad-leaved vegetation (BL), *Pinus* monoculture (PN), and enriched *Pinus* vegetation (PNEN) based on vegetation diversity.

According to the Eigen values of the principle component analysis carried out to determine the variation among ecosystems based on the diversity of arthropod families, ecosystem AQ differed prominently from other ecosystems. This was because of the higher abundance of arthropods of orthopteran families such as Tettigonidae and Cicadellidae, coleopteran families such as Lampyridae, hemipteran families including Gerridae and Diapsididae, and the lower abundance of several arachnid families including Pholcidae, Uloboridae, Scytodidae, and Clubionidae (Figure 6). Ecosystem BL, characterized by the high abundance of arthropods of Family Tettigonidae and low abundance of arthropods of families Curculionidae also showed a clear separation from other ecosystems (Figure 6). Based on their arthropod diversity, ecosystems PN and PNEN showed greater similarity to each other than to the other two ecosystems. This is primarily due to the high abundance of arthropods of families Curculionidae, Uloboridae, and Pscheridae in PN and PNEN (Figure 6). The PCA conducted based on vegetation diversity, the four ecosystems showed a highly prominent divergence (Figure 7).

## 4. Discussion

The principal focus of this work was to determine the dependence of arthropod diversity on vegetation diversity across four different ecosystems which are located close to each other in a mid-elevation humid tropical environment. Despite being a short-term study covering a limited area, our results provide important preliminary answers to the research questions that we posed at the beginning. Our results demonstrate significant variations in arthropod diversity (in terms of the Shannon-Wiener index) and their species richness among the four ecosystems, with the two *Pinus* based ecosystems (PN and PNEN) having significantly lower diversity than the two relatively un-disturbed ecosystems, the broad-leaved evergreen forest (BL) and the aquatic-based environment (AQ). However, our results only partially confirmed the expected positive relationship between arthropod diversity and vegetation diversity, which had been demonstrated in previous work [2, 5, 22, 23]. In agreement with such work, our results also showed that arthropod diversity across the three ecosystems which had not experienced direct and recent human intervention showed positive linear dependence on the vegetation diversity across the three habitats.

However, the most important finding of our work is the absence of an increase in arthropod diversity with the increased vegetation diversity in the enriched *Pinus* ecosystem even after two decades from artificial enrichment planting. This is in disagreement with the past work which had demonstrated the positive relationship between arthropod- and vegetation diversity. In fact, such studies had involved artificial manipulation of the vegetation diversity and productivity via sowing seeds of different numbers of plant species [2] application of fertilizer at different rates [14] and genetic hybridization [22]. Contrary to the observed increases in arthropod diversity in the above work, enrichment planting and consequent increases in vegetation diversity, and most likely the net primary productivity (not measured in our work), in the enriched *Pinus* ecosystem had not increased the arthropod diversity. This finding indicates that the resource specialization hypothesis (RSH) has a greater influence than the more individual hypothesis (MIH) in determining the arthropod diversity in the humid, tropical climate of the Hanthana mountain range. As the RSH is based on a majority of the arthropod species in a community showing host-specificity, it is likely that arthropod species specific to the introduced indigenous tree species have not been able to colonize and establish in the enriched *Pinus* ecosystem. This is plausible when we take into consideration the fact that many of the introduced tree species are not natives of the Hanthana mountain range. It is also possible that even after two decades, the environmental conditions within the enriched *Pinus* ecosystem are not conducive to support a broad diversity of arthropods species. Our observation that the dominant arthropod species in PNEN was a species of family Formicidae supports this explanation because arthropods of family Formicidae (ants) are a group which can adapt to any environmental condition rapidly.

Our observations that the dominant arthropod species in the aquatic and broad-leaved evergreen forest environments are spider species confirms the fact that those ecosystems are subjected to minimum human intervention, because spiders are extremely sensitive to unfavorable environmental conditions (e.g. pesticides and inorganic fertilizer). This observation agrees with results obtained from parallel studies where highest species richness, species abundance, individual abundance, and Shannon–Wiener index of spiders were recorded from natural forests of the Hanthana mountain range [21]. The dominance of spider species in two ecosystems having the highest plant diversity supports the conclusion of Haddad et al. [2] that greater vegetation diversity is conducive to development of a predator-dominated arthropod community.

The high sensitivity of spiders to unfavourable environmental factors could be a reason for the spider population to be very low in the enriched *Pinus* ecosystem as it is an ecosystem exposed to inorganic fertilizer when the land was artificially enriched. The lower abundance of other arthropod species in ecosystems that are dominated by spider species can be due to the fact that spiders are voracious predators of insects. Nevertheless, this observation supports the fact that increased arthropod herbivores with increasing plant diversity increases diversity of arthropods at higher trophic levels, thus leading to a greater diversity of predators [23].

The dominant arthropod species in the *Pinus* ecosystem is *Dicaldispa* spp. commonly known as rice hispa. It is a pest infesting plants of the Family Poeceae (Grasses). This is plausible because the plant which shows the highest abundance in this environment is *Panicum maximum* (Family Poaceae), which is present as an undergrowth of the *Pinus* monoculture. Rice hispa could have been introduced to this ecosystem from the rice fields which are located in the neighboring areas.

We acknowledge the limitations of this study in that our findings are based on a single round of measurements on a limited number of small plots. Therefore, our findings can only be considered as preliminary and need validation through a longer-term study involving a greater number of observational plots. The conclusions of this study can be taken as a baseline for the status of biodiversity in the Hanthana mountain range during the post-monsoonal dry season. Conducting future research to test whether these conclusions stand their ground in other seasons of the year as well, is recommended. Further research based on the foraging patterns and host-species relationships of the arthropods identified from these ecosystems is another promising research area that could explain the plant-arthropod interrelationships and community structure better. Nevertheless, we also point out that all four ecosystems that we used for sampling in our study have been free from any direct manipulations such as fertilizer application, selective thinning or enrichment. It has been so for the enriched *Pinus* ecosystem during the last 1 ½ decades. By using four different sampling methods, we have sampled the arthropods across a reasonable range of heights and depths. Furthermore, to the best of our knowledge, this is the only study that has been carried out so far in the Hanthana mountain range, covering the overall biodiversity of arthropods in general, and their interaction with the diversity of plants. Therefore, our results constitute an important addition to the very limited knowledge based on the dependence of arthropod diversity on vegetation diversity, especially in the humid tropical environments which the Hanthana mountain range represents. The regression models introduced from our work could also lay the foundation for more extensive studies aimed at describing community structures, interspecific relationships, and finding pathways of conserving and enriching the biodiversity of this specific region, which is under severe pressure from human interference and urbanization.

## 5. Conclusion

This study concludes that when human intervention is minimal in an ecosystem, the arthropod diversity is linearly dependent on the plant diversity of that ecosystem. However, in an ecosystem where human intervention is high, even though the plant diversity is increased artificially, the arthropod diversity does not increase accordingly.

## Figures and Tables

**Table 1:** Variation of arthropod and vegetation diversity in the ecosystems selected for the study.

Ecosystem	Arthropods			Vegetation		
	Shannon–Wiener index	Species richness	Evenness	Shannon–Wiener index	Species richness	Evenness
Aquatic	2.641^a^	16.33^a^	0.972^a^	1.810^b^	22.33^a^	0.601^b^
Broad-leaved	2.444^a^	12.67^ab^	0.983^a^	1.871^b^	15.00^a^	0.698^b^
*Pinus*	1.411^b^	5.00^c^	0.974^a^	0.879^c^	21.67^a^	0.286^c^
*Pinus* enriched	1.350^b^	9.67^bc^	0.635^b^	2.614^a^	19.67^a^	0.884^a^
CV (%)	26.99	43.90	8.47	22.38	32.68	18.48
*p* > *F*	0.057	0.0005	0.0032	0.011	ns	0.0038

Along each column, means with the same letter are not significantly different at *p* = 0.1 based on least significant difference. ns–non-significant at *p* = 0.05. CV–coefficient of variation.

## Data Availability

All data used in this research are included in the text.

## References

[1] Burkle L. A., Souza L., Genung M. A., Crutsinger G. M. (2013). Plant genotype, nutrients, and G × E interactions structure floral visitor communities. *Ecosphere*.

[2] Haddad N. M., Crutsinger G. M., Gross K., Haarstad J., Knops J. M., Tilman D. (2009). Plant species loss decreases arthropod diversity and shifts trophic structure. *Ecology Letters*.

[3] Ebeling A., Hines J., Hertzog L. R. (2018). Plant diversity effects on arthropods and arthropod-dependent ecosystem functions in a biodiversity experiment. *Basic and Applied Ecology*.

[4] Basset Y., Cizek L., Cuénoud P. (2012). Arthropod diversity in a tropical forest. *Science*.

[5] Castagneyrol B., Jactel H. (2012). Unraveling plant–animal diversity relationships: a meta‐regression analysis. *Ecology*.

[6] Underwood N., Inouye B. D., Hambäck P. A. (2014). A conceptual framework for associational effects: when do neighbors matter and how would we know?. *The Quarterly Review of Biology*.

[7] Barbosa P., Hines J., Kaplan I., Martinson H., Szczepaniec A., Szendrei Z. (2009). Associational resistance and associational susceptibility: having right or wrong neighbors. *Annual Review of Ecology Evolution and Systematics*.

[8] Bernays E., Graham M. (1988). On the evolution of host specificity in phytophagous arthropods. *Ecology*.

[9] Price P. W., Bouton C. E., Gross P., McPheron B. A., Thompson J. N., Weis A. E. (1980). Interactions among three trophic levels: influence of plants on interactions between insect herbivores and natural enemies. *Annual Review of Ecology and Systematics*.

[10] Hooper D. U., Chapin F. S., Ewel J. J. (2005). Effects of biodiversity on ecosystem functioning: a consensus of current knowledge. *Ecological Monographs*.

[11] Crutsinger G. M., Collins M. D., Fordyce J. A., Gompert Z., Nice C. C., Sanders N. J. (2006). Plant genotypic diversity predicts community structure and governs an ecosystem process. *Science*.

[12] Johnson M. T., Lajeunesse M. J., Agrawal A. A. (2006). Additive and interactive effects of plant genotypic diversity on arthropod communities and plant fitness. *Ecology Letters*.

[13] Haddad N. M., Tilman D., Haarstad J., Ritchie M., Knops J. M. (2001). Contrasting effects of plant richness and composition on insect communities: a field experiment. *The American Naturalist*.

[14] Siemann E., Tilman D., Haarstad J., Ritchie M. (1998). Experimental tests of the dependence of arthropod diversity on plant diversity. *The American Naturalist*.

[15] Prather R. M., Castillioni K., Welti E. A. R., Kaspari M., Souza L. (2020). Abiotic factors and plant biomass, not plant diversity, strongly shape grassland arthropods under drought conditions. *Ecology*.

[16] Woodcock B. A., Redhead J., Vanbergen A. J. (2010). Impact of habitat type and landscape structure on biomass, species richness and functional diversity of ground beetles. *Agriculture, Ecosystems & Environment*.

[17] Brose U. (2003). Bottom-up control of carabid beetle communities in early successional wetlands: mediated by vegetation structure or plant diversity?. *Oecologia*.

[18] Ashton P. M., Gamage S., Gunatilleke I. A., Gunatilleke C. V. (1997). Restoration of a Sri Lankan rainforest: using Caribbean pine Pinus caribaea as a nurse for establishing late-successional tree species. *Journal of Applied Ecology*.

[19] Knops J. M., Tilman D., Haddad N. M. (1999). Effects of plant species richness on invasion dynamics, disease outbreaks, insect abundances and diversity. *Ecology Letters*.

[20] Wyss E. (1996). The effects of artificial weed strips on diversity and abundance of the arthropod fauna in a Swiss experimental apple orchard. *Agriculture, Ecosystems & Environment*.

[21] Chathuranga W. G., Ranawana K. B. (2017). Spider fauna (arachnida: araneae) of upper Hanthana mountain area, central Sri Lanka. *Indian Journal of Arachnology*.

[22] Bangert R. K., Turek R. J., Martinsen G. D., Wimp G. M., Bailey J. K., Whitham T. G. (2005). Benefits of conservation of plant genetic diversity to arthropod diversity. *Conservation Biology*.

[23] Siemann E. (1998). Experimental tests of effects of plant productivity and diversity on grassland arthropod diversity. *Ecology*.

